# The Demographic and Health Surveys Faculty Fellows Program: Successes, Challenges, and Lessons Learned

**DOI:** 10.9745/GHSP-D-20-00318

**Published:** 2021-06-30

**Authors:** Wenjuan Wang, Shireen Assaf, Thomas Pullum, Sunita Kishor

**Affiliations:** a The Demographic and Health Surveys Program, ICF, Rockville, MD, USA.

## Abstract

Primarily funded by the United States Agency for International Development (USAID), the Demographic and Health Surveys (DHS) Faculty Fellows Program is designed to strengthen the institutional capacity of universities in low- and middle-income countries to use and analyze DHS data. This article provides an overview of the program's evolution and its current form; presents successes and impacts; and discusses challenges, lessons learned, and potential further directions.

Initiated in 2008, the Fellows Program underwent several rounds of improvement. The current model involves a competitive team application process, 2 intensive in-person workshops, completion of a working paper of publishable quality, and the transfer of analysis skills learned during the fellowship to students and colleagues at home universities, all completed over 1 year. Since 2011, the Fellows Program has trained 152 researchers from 45 universities in 25 countries in Africa, Asia, and the Middle East. The program has not only strengthened individual skills in conducting research with data from large surveys but also substantially increased institutional capacity to analyze DHS data through fellows' capacity-building activities at their home universities. These successes have happened despite the many and continuing challenges related to fellows' diverse backgrounds, experience, skills, language barriers, logistical difficulties in organizing in-person workshops in fellows' countries, and sometimes fellows' varying levels of commitment.

With continuous support from USAID, the program hopes to expand to more universities and countries, especially francophone countries. Overall, the DHS Faculty Fellows Program has proven to be highly successful and provides a model for other groups aiming to strengthen capacity for data use and research in low- and middle-income countries.

## Abstract (precis)

Since 2011, the Demographic and Health Surveys (DHS) Faculty Fellows Program has strengthened individual skills in conducting research with data from large surveys and increased institutional capacity to analyze DHS data through fellows' capacity-building activities at their home universities. The lessons learned can inform models for strengthening capacity in analyzing and using data in low- and middle-income countries.

## Teaser

**Key Findings:**

- The Demographic and Health Surveys (DHS) Faculty Fellows Program has trained 152 researchers from 45 universities in 25 countries in Africa, Asia, and the Middle East—demonstrating that it is possible to improve research and analysis skills in low- and middle-income countries through a short-term targeted program.
- The program has not only strengthened individual skills in conducting research with data from large surveys but also substantially increased institutional capacity to use and analyze complex survey data through the fellows' cascading of capacity-strengthening activities at their home universities.

**Key Implications:**

- Continued investment is needed to strengthen the countries' capacity to use complex survey data for their policy and program purposes.
- The DHS Faculty Fellows Program provides a successful model that other international development programs can use to strengthen capacity in analysis and use of data in low- and middle-income countries.
- Capacity-strengthening programs should engage local institutions to be more effective and sustainable.

## INTRODUCTION

The Demographic and Health Surveys (DHS) Program is a United States Agency for International Development (USAID)-funded project that assists low- and middle-income countries with the implementation of nationally representative household surveys that collect data on population, health, and nutrition and with the analysis and dissemination of these data. Since 1984, the DHS Program has implemented more than 400 surveys in 90 countries and is a major source of reliable data that inform national and international population and health policies and programs. In addition to technical assistance in data collection, the DHS Program is committed to strengthening the capacity of host-country partners all along the survey continuum, from survey and sample design to data analysis, dissemination, and use. The DHS Faculty Fellows Program is a major component of the DHS Program's long-standing commitment to increasing host-country capacity to use DHS data. Its primary objective is to strengthen the institutional capacity of universities in participating countries to understand the DHS microdata and use them to conduct complex analyses to answer policy- and program-relevant questions. The DHS Faculty Fellows Program seeks to create sustainable in-country capacity by training university faculty whose role is to educate students who will be the country's future policy makers, program managers, and researchers. Although the Fellows Program trains individuals, 2 key elements help to cascade the learning to the fellows' home universities. First, individual faculty are enrolled in the program as a team with other faculty from the same university, allowing for reinforcement of learning. Second, the terms of the Fellows Program require that on completion, the trained faculty will implement self-designed activities to transfer their learning to others within the university. To ensure institutional commitment to these program requirements, fellows' applications must include evidence of permission from their university authorities.

This article provides an overview of the program's evolution; describes its current form; presents successes and impacts; and discusses challenges, lessons learned, and potential further directions. The DHS Faculty Fellows Program provides a valuable model for strengthening capacity for data analysis and research in low- and middle-income countries. Experience and lessons gained from this program could also inform global capacity-strengthening programs in areas beyond research and data analysis.

## EVOLUTION OF THE FELLOWS PROGRAM

**FIGURE 1:**
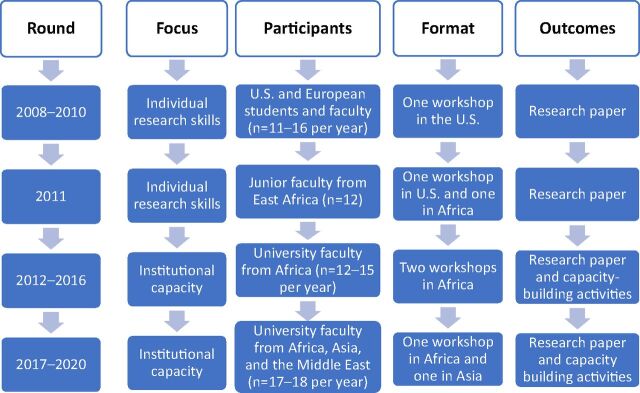
Evolution of the Demographic and Health Surveys Faculty Fellows Program

The DHS Fellows Program originated in 2008 and went through several major shifts in the course of its development (Figure 1).

### Initiation Approach: Train Individuals to Produce Academic Papers

The original DHS Fellows Program was designed to focus on building individual skills to prepare academic papers based on DHS data. The first 2 rounds of the Fellows Program, in 2008–2009 and 2009–2010, included a competitive application process, with a journal-quality research paper as the final deliverable, mentoring of each participant by a member of the DHS technical staff, and a US workshop. Although it was open to qualified candidates from any country, the applicants were primarily graduate students and junior faculty from US and European universities. Fellows received remote guidance and support, mainly from their DHS mentor, on formulating research questions, analyzing data, preparing an analysis plan, and writing the final paper. Toward the end of each round, all fellows came to the DHS headquarters in Maryland, USA, for a 2-week workshop, during which they received intensive one-on-one assistance from their mentors and other DHS staff to finalize their research papers.

The first 2 rounds successfully trained many researchers and provided valuable lessons for future rounds of the program. Participant feedback emphasized the need for an additional workshop at the beginning of the program, which would provide more formal training on the fundamentals and correct use of DHS data before fellows began their own analysis. Two workshops could maximize the learning experience and facilitate the production of high-quality papers. Although these rounds of the program included some participants from low- and middle-income countries, there was no exclusive focus on these countries where there is a great need and a high demand for capacity strengthening in data analysis and use.

### Shift Focus to University Faculty in Low- and Middle-Income Countries

Based on the lessons from the first 2 rounds and the recognition that the format did not fulfill the DHS Program mandate to build capacity in DHS host-countries, the Fellows Program was completely redesigned in 2011 to target universities in low- and middle-income countries. In this iteration, the program focused specifically on junior faculty, who would have many years to train students and contribute to their institutions. In this round, DHS limited the call for applications to several East African countries that had implemented multiple rounds of DHS surveys. Two workshops were provided for the participants: the first was early in the fellowship to provide a solid foundation for DHS data use and instruction, and the second was several months later, allowing sufficient time for fellows to work on their research papers. The first workshop was held in Kenya that year and the second at DHS headquarters. The 2011 program represented 10 universities in 6 East African countries. Most fellows successfully completed a paper that was subsequently published in the DHS Working Paper series.

Although the first 2 rounds of the DHS Fellows Program trained many researchers, the program was redesigned in 2011 to focus on university faculty in DHS host-countries.

### Add Emphasis on Strengthening Institutional Capacity

Although the 2011 round of the program did consider institutional capacity building—for example, by focusing on junior faculty who may have many years to “give back” to their universities—it was not until the 2012 round that institutional capacity strengthening became a key focus of the program. One modification to this end was to replace applications from individuals with applications from 3-person teams from the same university. The 3-member faculty teams were expected to work together to develop their research proposal, complete a research paper, and develop and implement a capacity-building plan for their home universities. This modified approach was aimed at helping increase institutional capacity in data analysis and research through training more than 1 faculty at a time so that team members could reinforce the learning, and by requiring that the training be cascaded to other faculty and students in each participating university. To ensure successful implementation of the capacity-strengthening plan at the home universities, each team was required to include a senior faculty member who would likely have a greater influence on curriculum development and research activities in the department.

### Expand the Program From Africa to Asia and the Middle East

For the first few years, the program primarily focused on countries in sub-Saharan Africa, but in 2017 it was expanded to Asia and the Middle East. The expansion to these regions was motivated by DHS statistics on the number of DHS datasets downloaded by country that indicated limited requests for and use of DHS data in these regions (except for India), despite the availability of multiple surveys in many countries. In addition, analysis of data from DHS's tracking of journal articles using DHS data also showed that very few papers had been written by authors from South and Southeast Asia (excluding India). These data suggested a need for capacity building in DHS data use and analysis in this region.

## THE CURRENT MODEL

The current model of the program is a result of the major modifications discussed above and many other improvements over the years. The current model has more streamlined processes for participant selection, 2 in-person training workshops, and postworkshop activities.

The current model of the program is a result of previous modifications, including improvements to participant selection, training workshops, and postworkshop activities.

### Selection of Participants

In October or November of every year, DHS issues a call for applications from universities in the targeted countries. These countries are selected based on the availability of recent DHS data and the existence of universities offering academic programs in population and health. Countries targeted can vary every year; priority is given to those that have never participated or have had low participation in previous program rounds. Other important considerations relate to the amount and type of funding available for the program. The program typically selects 5 or 6 teams, each composed of 3 faculty members, on a competitive basis using criteria such as quality of the research proposal, applicants' experience and baseline skills in data analysis, suitability of the proposed capacity-building activities to the applicants' university, country diversity, and gender balance. As the competitive selection process may result in applicants from resourceful universities having a better chance to be selected, the program carefully assesses the diversity of selected universities to reduce repeating participation from the same universities.

### First Training Workshop

The program provides in-person training through 2 workshops led by DHS technical staff. These 2-week workshops are usually held in the fellows' home countries so that it is possible to involve DHS implementing agencies and other stakeholders, including the USAID Mission. Fellows also receive intensive remote guidance and feedback before and after workshops.

Before the first workshop, an online preworkshop assignment prepares the fellows for the training. This includes downloading the required DHS datasets and completing a short course on the objectives and coverage of the DHS Program. The online platform also includes other important learning resources and allows fellows to interact with DHS facilitators. The training curriculum for the first workshop prepares fellows to analyze DHS data from their respective countries. Topics include DHS questionnaires, DHS recode data files and variables, principles of survey sampling and weighting, how to account for complex survey design in the analysis, use of statistical software (usually Stata) for analysis, dataset merging, variable recoding, and multivariate regression analysis. For each topic, the concepts are presented and then enhanced by participatory exercises in Stata. The curriculum also includes discussions on research conceptualization, preparation of a tabulation plan, scientific writing, research ethics, and citation software. During this workshop, fellows' teams also work with DHS facilitators to refine the design of their research projects and to conduct preliminary data analysis. Research topics are related to reproductive health, family planning, maternal and child health, HIV, nutrition, and gender issues. Pre- and posttests are used to evaluate the effectiveness of the training.

### Second Training Workshop

Between the first and second workshops, while back at their home university, fellows prepare and submit a draft working paper, for which they receive extensive reviews and comments from the DHS staff. The second workshop, which takes place approximately 2 months after the first workshop, concentrates on revising and finalizing the working paper draft, to ensure that the research meets publishable quality standards. By the end of the second workshop, most teams are nearly finished with their revised paper. During this workshop, fellows are also introduced to more advanced topics such as estimation of fertility and child mortality using DHS data and analyzing DHS calendar data, as well as more advanced statistical methods such as multilevel modeling, survival analysis, and decomposition analysis. These topics are selected based on fellows' interests and skills. All the training materials are shared with the fellows for their own teaching and capacity-building activities. DHS is also currently putting these training materials in an online repository to benefit more DHS data users. For each workshop, a final evaluation is conducted to solicit feedback on training quality and usefulness.

### Posttraining Activities

In addition to completing a research paper, fellows are required to design and implement a series of capacity-building activities at their home universities to share their learning with students and faculty colleagues. Activities typically include integrating DHS data into their teaching curriculum, department seminars, and research meetings to increase awareness of DHS data; mentoring graduate students to use DHS data in theses or dissertations; and conducting DHS data analysis workshops for students and faculty. Fellows conduct these activities primarily with support from their home university. The teams are required to submit 2 reports on the implementation and results of these capacity-building activities.

## PROGRAM SUCCESSES AND IMPACTS

### High-Quality Research Published in Peer-Reviewed Journals

**FIGURE 2:**
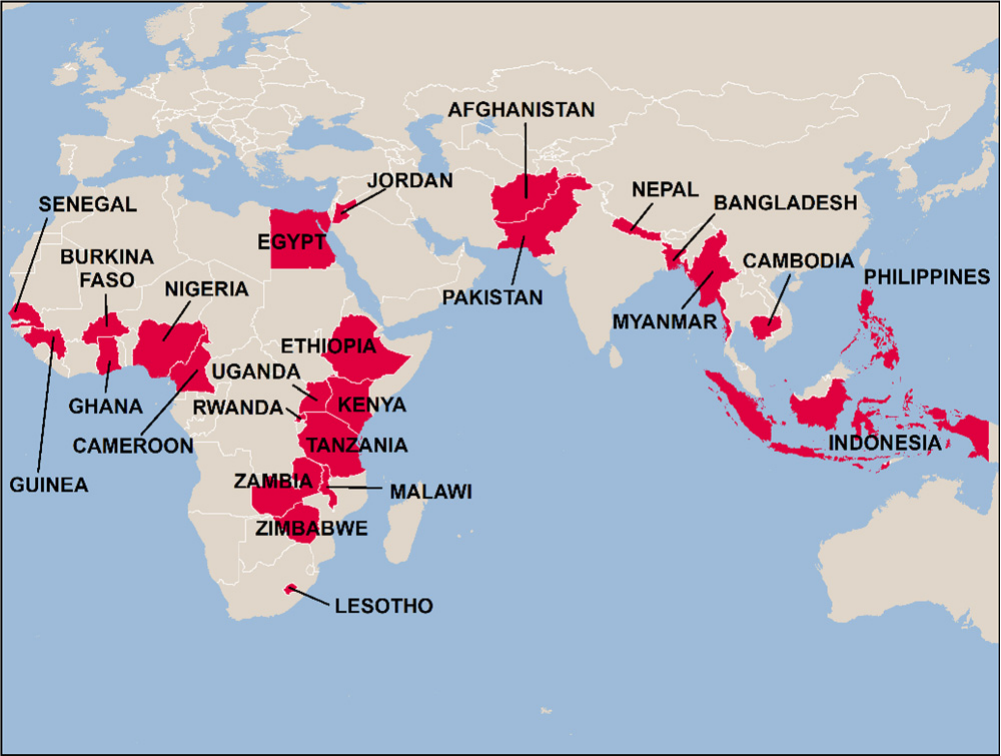
Countries Included in the Demographic and Health Surveys Fellows Program During 2011–2020

Since 2011, the Fellows Program has trained 152 researchers from 45 universities in 25 countries in Africa, Asia, and the Middle East (Figure 2). The program has increased individual skills in conducting research with data from large surveys, as indicated by 57 high-quality research papers published as working papers on the DHS website. Most of these papers, after revisions, have been published in peer-reviewed journals (see Supplement) such as *Studies in Family Planning*, *PLoS One*, *BMC Pregnancy and Childbirth*, *African Population Studies*, *Reproductive Health*, and the *International Journal of Population Research.*

Since 2011, the Fellows Program has trained 152 researchers from 45 universities in 25 countries in Africa, Asia, and the Middle East.

Beyond their fellowship papers, fellows have continued to use DHS data in their own research, and many have published other research based on DHS data after they participated in the program. In 2016 and 2017 alone, 15 papers were published by former fellows. Many fellows have presented their research findings based on DHS data at national and international scientific meetings. These post-fellowship activities have been self-motivated and have not received financial support from the DHS Program.

### Increased Institutional Capacity in Data Analysis and Research

In addition to producing sound research based on DHS data, the Fellows Program has substantially increased institutional capacity to analyze DHS data through the fellows' capacity-building activities at their home universities. Many activities continue after the end of the fellowship. Fellows are particularly successful when there are other fellows from previous cohorts working at the same university. For example, teams of Nigerian fellows from Obafemi Awolowo University who participated in different rounds of the Fellows Program (2012, 2014, and 2016) have worked together and conducted annual training on DHS data analysis since 2012. In 2014, they expanded the training beyond students and faculty in their university to other universities and nonacademic research institutions. Between 2012 and 2016, they trained over 100 participants from a variety of universities and organizations in Nigeria to use DHS data. Fellows from several cohorts at Makerere University in Uganda also collaborated to expand capacity-building activities and conduct research based on DHS data. By involving participants from government agencies and local organizations, some fellows have built a network with local stakeholders for future collaboration and data analysis support to these organizations.

Fellows have written blogs on the impact of the Fellows Program on themselves and their university. We present 2 quotes below from the fellows.

*Since 2018, I published 2 journal articles and presented 2 oral presentations at the 10th and 11th International Conference on Public Health among Greater Mekong Sub-Regional Countries. In addition, 3 of my MPH students prepared their proposals using DHS data this year. Myanmar is now realizing the data quality and accuracy of DHS indicators, so, not only academicians and students but also program managers and policymakers are using DHS indicators in relevant situations*. —Fellow from Myanmar 1 year after completing the 2018 program

*Thanks to the Fellows Program, we are better equipped to use this data in other work and have shared it with our colleagues during our capacity-building activities. Some colleagues are already hoping to participate in future Fellows Program or other DHS workshops. This program not only allowed us to better understand the DHS surveys, but also make in-depth statistical analyses and to use DHS data to write analysis reports.* —Senegal team in the 2019 program

### Continued Impact on Fellows and Their Institutions

**FIGURE 3:**
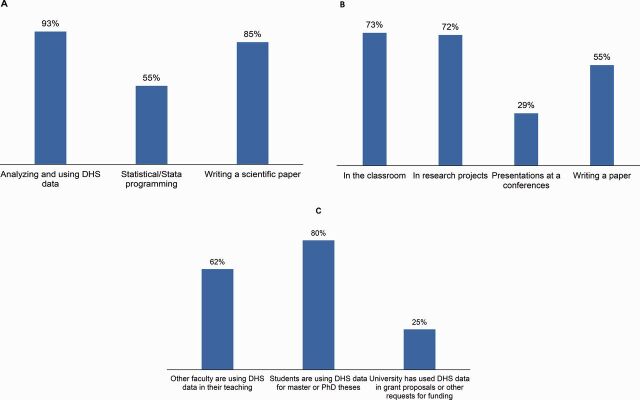
Result of the Demographic and Health Surveys Fellows Follow-up Surveys, 2015–2018. (A) Skills learned or strengthened during the Fellows Program that were used since finishing the program, 2015–2018 (n=62); (B) Contexts in which DHS data were used by fellows after finishing the program, 2015–2018 (n=62); (C) Ways that fellows' universities have benefited from their participation in the Fellows Program, 2015–2018 (n=62)

The Fellows Program has shown continued impact on fellows and their institutions after the fellowship ends. We analyzed data from a follow-up survey conducted 6 months after the conclusion of the program with the 2015, 2016, 2017, and 2018 cohorts. The response rate was 100% from all cohorts except the 2018 cohort, for which it was 83%. Figure 3 highlights some survey results. Most fellows used their skills in analyzing and using DHS data (93%) and writing a scientific paper (85%) after finishing the program. Fellows have continued to use DHS data in different contexts, including teaching and in research projects. Almost all fellows reported that their universities have benefited from their participation in the Fellows Program through their own capacity-strengthening activities. Their participation in the Fellows Program increased the use of DHS data in their own and colleagues' teaching curricula and research projects, as well as in their students' theses or dissertations.

The Fellows Program has shown continued impact on fellows and their institutions after the fellowship ends.

### Source of South-to-South Consultants for the DHS Program

The Fellows Program has also been an important source of south-to-south consultants for other analysis workshops of the DHS Program. Alumni fellows have a combination of technical skills gained through the program and considerable experience with teaching—their main occupation. Former fellows have often been invited back as co-facilitators in many types of DHS workshops, including subsequent fellows workshops. By 2019, 20 fellows had been invited back to co-facilitate fellows workshops and other DHS data use and analysis workshops, as well as to co-author reports that further analyze their country's DHS data. Former fellows had very positive feedback on the value of facilitation for their careers and further improvement of their analytical and research skills.

## CHALLENGES AND LESSONS LEARNED

While achieving many successes, the DHS Fellows Program also faces challenges related to fellows' diverse backgrounds, experience, and skills; logistical difficulties in organizing in-person workshops; language barriers; and fellows' varying levels of commitment.

While achieving many successes, the DHS Fellows Program also faces challenges.

### Diversity of Backgrounds, Experience, and Skills

Teams are selected through an open competitive process that involves rigorous assessments of skills and experience based on applications. However, the baseline capacity of the participants can still vary greatly, particularly in prior knowledge and use of survey data, computer skills, familiarity with statistical software, and research ability. Most fellows have been junior faculty with a master's degree received from their home country and very limited previous experience working with large survey datasets and complex analytical methods. In addition to various skill levels, fellows come from a wide range of research backgrounds, including demography, public health, medicine, social sciences, psychology, and health systems. While this diversity has its advantages in expanding the fellows' exposure to various research areas, it also creates challenges for teaching and mentoring the fellows. The DHS facilitators must engage different interests and accommodate varying skill levels during lectures and hands-on work, while still ensuring that all content is covered within a fixed amount of time. Facilitators must provide one-on-one intensive mentorship to teams with limited skills. At times, major adjustments to fellows' research proposals are required to narrow their original scope or to shift to simpler analytical methods if the team has limited capacity. Teams with more experience are encouraged to apply more advanced methods in their research. Cofacilitators, typically former fellows, provide extra assistance during the limited time available. They also liaise between the DHS facilitators and fellows and are especially helpful in bridging differences in culture and primary language.

Despite the efforts to encourage women's participation in the Fellows Program, for example, making gender balance an explicit selection consideration, the proportion of female fellows is low, constituting less than 40% in 7 rounds between 2011 and 2020. This proportion is consistent with the sex ratio among all applicants, reflecting the underrepresentation of women in academia in low- and middle-income countries.

### Logistical Challenges

The in-person workshops for fellows are usually held in the home countries of the participating teams, but the selection of the workshop locations must consider accessibility in terms of flights and visas for all the teams. Since the program was expanded to include scholars from both Asia and Africa, the program has aimed to have 1 workshop in Asia and 1 in Africa. Due to the wider geographic dispersion of DHS countries in Asia, it is more difficult to identify an optimal location in Asia than in Africa. There are fewer options for a location with reasonable flight times and no overnight stays during travel. At times, visa accessibility becomes a very critical factor in choosing where to have the workshops, especially when the program includes countries with limited visa access to other countries. Sometimes fellows must travel to a neighboring country because the country for which they need a visa does not have an embassy in their own country. Countries that do not require a visa or that allow for an e-visa are advantageous for workshops, although the travel cost is sometimes higher or the itineraries not as convenient. Therefore, the choice of location is determined by a balance of several factors. For example, 1 workshop was held in Thailand, a non-DHS country, because it offered better visa access, was less expensive, and had more convenient flights than any of the fellows' host countries. This selection, however, did not reflect the goal of having workshops in countries with DHS surveys to better engage local stakeholders.

### Language Barriers

To date, English has been the primary language for the Fellows Program, even though it is not necessarily the primary language in the fellows' countries. For example, although English may be one of the official languages in East African countries, Swahili is more widely spoken than English. The use of English as the program's medium can be a language barrier for some fellows. Recognizing this, DHS uses several measures to overcome language barriers. These include, but are not limited to, making the teaching materials clear, simple, and concise; always sharing presentations and statistical software programs with fellows; providing exercises for fellows to practice on their own time; and encouraging fellows to ask questions during presentations. The training curriculum has been translated into other languages such as French and Arabic and shared with fellows. The program enlists co-facilitators who play an important role in helping the fellows with language challenges. Co-facilitators are former fellows who have an especially good understanding of the training materials. They have been very helpful in offering supplementary explanations, often in their own language. Fellows are generally active in discussions and ask questions during presentations. Given the preparation and assistance, language has not been a major problem in effectively communicating and has not been a barrier to understanding training materials.

Writing a scientific paper in English is challenging for almost all fellows—as it is for any non–English-speaking researcher. There is no easy way to overcome this hurdle despite the sharing of materials on writing a scientific paper that include a working paper template. All fellows' papers are professionally edited, and fellows review the edits before publication.

### Fellows' Commitment

Fellows' commitment to the requirements and aims of the program is critical to its success. Such commitment includes being responsive to travel arrangements, full participation in the 2 workshops, and timely submission of deliverables. To ensure fellows' participation, especially their travel to the workshops, fellows are required to submit a letter of approval from their universities, assuring permission to participate. DHS also issues a contract with each fellow at the beginning of the fellowship with detailed specifications of activities, deliverables, and timelines, as well as penalties for noncompliance. On a few occasions in both regions, some fellows have been unable to attend the second workshop due to unexpected work commitments. These fellows are usually the senior members of teams. Senior members are helpful in overseeing teamwork and ensuring capacity-building activities at the home university, but they face special challenges in committing the time required to complete the program. They may work remotely with other team members to complete the work, but their absence from workshops has a negative effect on productivity and morale. Thus, DHS is now more cautious about enrolling fellows with high-level administrative positions.

## FUTURE OF THE PROGRAM

With support from USAID, the DHS Program plans to continue the current model of the Fellows Program with further improvements where possible. The program will continue to include a mix of scholars from Asia and Africa, a feature that has received very positive feedback from participants since it increases collaboration between the universities and fellows in the 2 regions. The program is considering expansions in several areas.

The DHS Program plans to continue the current model of the Fellows Program with further improvements where possible.

### Expanding to Francophone Countries

We hope to expand the program to more countries and universities, especially, more francophone countries. The use of English has limited our ability to include francophone countries in the past, but it has not prevented it entirely. Our experience with including teams from Burkina Faso and Senegal was successful because the teams had a strong background in public health or demography, had prior experience using DHS data, and had some, albeit limited, English skills. To facilitate their participation, DHS included bilingual facilitators during the workshops and provided all materials in French as well as English. In the 2020 round, more French-speaking countries were targeted in the call for applications and 3 French-speaking teams (from Burkina Faso, Cameroon, and Guinea) were accepted. The inclusion of non–English-speaking teams is an important expansion of the Fellows Program but does imply a need for increased funding to accommodate these changes.

### Expanding to Research Institutions

Expanding the program to include research institutions was another option that was explored, especially for countries that do not have universities with strong programs and faculty in relevant disciplines. In 2019, the DHS Program experimented with this option but received very few applications, none of which survived the review process. One concern about including research institutions is their limited potential for skill cascading because they do not usually teach students. Skill transfer is certainly possible among research colleagues in the same institution, but higher job turnover compared with universities may reduce the impact. If research institutions are included in future programs, they need to be carefully selected to take into account their potential for capacity-building activities, and their incentives and motivation for continued use of DHS data.

### Expanding the Use of Virtual Teaching

Almost all training materials have been delivered to fellows through the in-person workshops. Given technological advances, it is now possible to deliver some of the materials through online courses before workshops to reduce the duration of the in-person training. The current program requires fellows to complete an online prework assignment that provides basic preparation for the in-person training. The current prework could be expanded to include some sessions that are usually provided at the beginning of the workshop, such as introductory sessions on Stata, understanding DHS standard recode files, using the DHS recode manual, and finding variables in DHS data files. Virtual learning has its constraints including limited inter-team interactions and requirements for reliable internet connectivity, which is not always possible in many fellows' countries. The second workshop of the 2020 Fellows Program was held online because of the COVID-19 pandemic. While it was found during this workshop that the virtual format was not effective in discussing complex technical topics, such change did not prevent the program from achieving its primary goal. Fellows increased their knowledge of DHS, mastered critical skills for analyzing DHS data, and successfully completed their research projects. Overall, training through a virtual platform can be a useful and economic supplement to in-person training if designed carefully.

Overall, the DHS Faculty Fellows Program has evolved to create and strengthen capacity in low- and middle-income countries in the often neglected but critical areas of data analysis and research. The program has been successful in supporting high-quality research during each round and stimulating subsequent trajectories of research productivity as evidenced by many peer-reviewed publications by former fellows. The inclusion of several countries in each round has promoted a better awareness of research issues and opportunities in different countries. The program has had a cascading effect by targeting university faculty and requiring them to develop mechanisms such as workshops and course modules within their home universities. The program has also strengthened the links that university faculty have with government agencies and USAID Missions, providing in-country expertise for the analysis of programs and policies. The experience and lessons learned in implementing the DHS Faculty Fellows Program provide a blueprint for other programs aiming to build capacity in analysis and use of data in low- and middle-income countries.

## Supplementary Material

20-00318-Wang-Supplement.pdf

## Notes

**Peer Reviewed**

**First published online:** May 21, 2021.

