# Supplementary material for: The Demographic and Health Surveys Faculty Fellows Program: Successes, Challenges, and Lessons Learned
Source: Glob Health Sci Pract. 2021 Jun 30;9(2):390–8. doi: 10.9745/GHSP-D-20-00318 (PMC8324205; doi:10.9745/GHSP-D-20-00318)
Supplement: 20-00318-Wang-Supplement.pdf [file 20-00318-Wang-Supplement.pdf]

Supplement to: Wang W, Assaf S, Pullum T, Kishor S. The Demographic and Health Surveys Faculty Fellows Program: successes, challenges, and lessons learned. *Glob Health Sci Pract*. 2021;9(2). https://doi.org/10.9745/GHSP-D-20-00318

Peer-reviewed journal papers published by DHS Fellows, 2011-2020

Fellows cohort	Title	Journal	Authors
2020	Social and individual factors associated with condom use among single youths: an analysis of the 2018 Cameroon Demographic and Health Survey	*Journal* of Biosocial Science	Jean-Robert Mburano Rwenge, Franklin Bouba Djourdebbe, and Emmanuel Ekambi Ekambi
2020	Disparities in cesarean section among women in Jordan: analysis of the 2017–2018 Jordan Population and Family Health Survey (JPFHS) data	*Journal of Maternal-Fetal Neonatal Medicine*	Israa Al-Rawashdeh, Ibrahim Kharboush, and Waqar Al-Kubaisy
2019	Determinants of nutritional status among children under age 5 in Ethiopia: Further analysis of the 2016 Ethiopia Demographic and Health Survey	*BMC Globalization and Health*	Zerihun Yohannes Amare, Mossa Endris Ahmed, and Adey Belete Mehari
2019	Predictors of modern contraceptive use and fertility preferences among men in Myanmar: Further analysis of the 2015-16 Demographic and Health survey	*International Journal of Community Medicine and Public Health*	May S. Aung, Pa P. Soe, and Myo M. Moh
2019	Uptake of intermittent preventive treatment in pregnancy for malaria: Further analysis of the 2016 Ghana Malaria Indicator Survey	*Journal of Public Health: From Theory to Practice*	Eugene Kofuor Maafo Darteh, Isaac Buabeng, and Clara Akuamoah-Boateng
2018	Facility delivery and postnatal care services use among mothers who attended four or more antenatal care visits in Ethiopia: Further analysis of the 2016 Demographic and Health Survey	*BMC Pregnancy and Childbirth*	Gedefaw Abeje Fekadu, Fentie Ambaw, and Seblewongiel Ayenalem Kidanie
2018	Factors affecting contraceptive use and unmet need among currently married women in Afghanistan: Further analysis of the 2015 Afghanistan Demographic and Health Survey	*Journal of Global Health Reports*	Sabawoon Ajmal, Anwar Idris, and Behzad Ajmal
2018	Factors influencing risky sexual behaviours among youths and adult men in Malawi	*Studies on Ethno-Medicine*	Beston B. Maonga, Tapiwa Sphiwe Gondwe, and Kennedy Machira

Supplement to: Wang W, Assaf S, Pullum T, Kishor S. The Demographic and Health Surveys Faculty Fellows Program: successes, challenges, and lessons learned. *Glob Health Sci Pract*. 2021;9(2). https://doi.org/10.9745/GHSP-D-20-00318

Fellows cohort	Title	Journal	Authors
2018	Feeding practices and nutritional status of children age 6-23 months in Myanmar: A secondary analysis of the 2015-16 Demographic and Health Survey	*PLoS One*	Kyaw Swa Mya, Aung Tin Kyaw, and Thandar Tun
2018	Influence of internal migration on the use of reproductive and maternal health services in Nepal: An analysis of the Nepal Demographic and Health Survey 2016	*PLoS One*	Naba Raj Thapa, Sunil Adhikari, and Pawan Kumar Budhathoki
2017	Low birth weight of institutional births in Cambodia: Analysis of the Demographic and Health Surveys 2010-2014	*PLoS One*	Chhorvann Chhea, Por Ir, and Heng Sopheab
2017	Ten years of traditional contraceptive method use in the Philippines: Continuity and change	*Studies in Family Planning*	Marquez, M. P., Kabamaln, M. M. and Laguna, E. P.
2016	Assessment of contraceptive use by marriage type among sexually active men in Nigeria	*International Quarterly of Community Health Education*	Asa, S. S., Titilayo, A. and Kupoluyi, J. A.
2016	Factors associated with postnatal care for newborns in Zambia: Analysis of the 2013-14 Zambia Demographic and Health Survey	*BMC Pregnancy and Childbirth BMC series*	Bupe B. Bwalya, Mulenga C. Mulenga, and James N. Mulenga
2015	Predictors of time-to-contraceptive use from resumption of sexual intercourse after birth among women in Uganda	*International Journal of Population Research*	Robert Wamala, Allen Kabagenyi, and Simon Kasasa
2015	Factors associated with changes in uptake of HIV testing among young women (aged 15-24) in Tanzania from 2003 to 2012	*Infectious Diseases of Poverty*	Michael Mahande, Rune Philemon, and Habib Omari of Kilimanjaro Christian
2015	Gender relations, sexual behaviour, and risk of contracting sexually transmitted infections among women in union in Uganda	*BMC Public Health*	Olivia Nankinga, Cyprian Misinde, and Betty Kwagala
2014	HIV knowledge and risky sexual behavior among men in Rwanda	*PanAfrican Medical Journal*	Etienne Rugigana, Francine Birungi, and Manasse Nzayirambaho
2014	Trends of modern contraceptive use among young married women based on the 2000, 2005, and 2011	*PLoS One*	Abebaw Gebeyehu Worku, Gizachew Assefa Tessema,

Supplement to: Wang W, Assaf S, Pullum T, Kishor S. The Demographic and Health Surveys Faculty Fellows Program: successes, challenges, and lessons learned. *Glob Health Sci Pract*. 2021;9(2). https://doi.org/10.9745/GHSP-D-20-00318

Fellows cohort	Title	Journal	Authors
	Ethiopian Demographic and Health Surveys: A multivariate decomposition analysis		and Atinkut Alamirrew Zeleke
2014	Why do women deliver at home? Multilevel modeling of Ethiopian National Demographic and Health Survey data	*PLoS One*	Henock Yebyo, Mussie Alemayehu, and Alemayehu Kahsay
2013	Household nucleation, dependency, and child health outcomes in Ghana	*Journal of Biosocial Science*	Kofi Awusabo-Asare, Samuel Kobina Annim, and Joshua Amo-Adjei
2013	Differences in risky sexual behaviors and HIV prevalence of circumcised and uncircumcised men in Uganda: Evidence from a 2011 cross-sectional national survey	*Reproductive Health*	Simon Peter Kibira, Elizabeth Nansubuga, and Nazarius Mbona Tumwesigye
2013	Does women's empowerment improve nutrition status among under-five children?	*International Journal of Physical Social Sciences*	Domitilla A. R. Bashemera, Grace Benedict, and Martha J. Nhembo
2013	Male circumcision and risky sexual behavior in Zimbabwe: Evidence from the 2010-11 Zimbabwe Demographic and Health Survey	*African Population Studies*	Antony Chikutsa, Shepard Mutsau, and Alfred C. Ncube
2013	Relationship between women's socioeconomic status and empowerment in Burkina Faso: A focus on participation in decision-making and experience of domestic violence	*African Population Studies*	Madeleine Wayack-Pambè, Bilampoa Gnoumou Thiombiano, and Idrissa Kabore
2013	Association between wanting circumcision and risky sexual behaviour in Zimbabwe: Evidence from the 2010–11 Zimbabwe Demographic and Health Survey	*Reproductive Health*	Antony Chikutsa, Shepard Mutsau, and Alfred C. Ncube
2012	Measuring the success of family planning initiatives in Rwanda: A multivariate decomposition analysis	*Journal of Population Research*	Dieudonne Ndaruhuye Muhoza, Pierre Claver Rutayisire, and Aline Umubyeyi

Supplement to: Wang W, Assaf S, Pullum T, Kishor S. The Demographic and Health Surveys Faculty Fellows Program: successes, challenges, and lessons learned. *Glob Health Sci Pract*. 2021;9(2). https://doi.org/10.9745/GHSP-D-20-00318

Fellows cohort	Title	Journal	Authors
2012	Male involvement in maternal health care as a determinant of utilization of skilled birth attendants in Kenya	*East African Medical Journal*	Judy Mangeni, Ann Mwangi, Samwel Mbugua, and Vincent Mukthar
2012	Use of maternal health care as a predictor of postpartum contraception in Nigeria	*African Population Studies*	Ambrose Akinlo, Adeleke Bisiriyu, and Olapeju Esimai
2012	Factors associated with modern contraceptive use among young and older women in Uganda: A comparative analysis	*BMC Public Health*	John Bosco Asiimwe, Patricia Ndugga, and John Mushomi
2011	Trends and determinants of undernutrition among young Kenya children: Kenya Demographic and Health Survey; 1993, 1998, 2003, and 2008-09	*Public Health Nutrition*	Peninah K. Masibo and Donald Makoka

Note: All authors in the papers are DHS Fellows.
